# The use of Leaf Surface Contact Cues During Oviposition Explains Field Preferences in the Willow Sawfly *Nematus oligospilus*

**DOI:** 10.1038/s41598-019-41318-7

**Published:** 2019-03-20

**Authors:** Patricia C. Fernández, Celina L. Braccini, Camila Dávila, Romina B. Barrozo, M. Victoria Coll Aráoz, Teresa Cerrillo, Jonathan Gershenzon, Michael Reichelt, Jorge A. Zavala

**Affiliations:** 1 Consejo Nacional de Investigaciones Científicas y Tecnológicas, Buenos Aires, Argentina; 2 INTA, Estación Experimental Agropecuaria Delta del Paraná. Paraná de las Palmas y Cl Comas s/n (2804), Campana, Buenos Aires Argentina; 3 Universidad de Buenos Aires, Cátedras de Química de Biomoléculas y Bioquímica, Facultad de Agronomía. Av. San Martín 4453, C1417DSE Ciudad Autónoma de Buenos Aires, Argentina; 4 INTA, Instituto de Recursos Biológicos, Centro de Investigación de Recursos Naturales, De los Reseros y Dr. Nicolás Repetto s/n (1686), Hurlingham, Buenos Aires Argentina; 5 Universidad de Buenos Aires, Departamento Biodiversidad y Biología Experimental, Facultad de Ciencias Exactas y Naturales, Instituto Biodiversidad y Biología Experimental y Aplicada, CONICET - UBA, Ciudad Autónoma de Buenos Aires, Argentina; 6 PROIMI-CONICET Biotecnología, Av. Belgrano y Pje, Caseros (T4001 MBV), Tucumán, Argentina and Facultad de Ciencias Naturales e IML, UNT, Miguel Lillo 205, San Miguel de Tucumán, Argentina; 7 Max Planck Institute for Chemical Ecology, Jena, Germany

**Keywords:** Chemical ecology, Forest ecology

## Abstract

After an insect herbivore has reached its host plant, contact cues from the leaf surface often determine host acceptance. We studied contact cues during oviposition behavior of a willow pest, the sawfly *Nematus oligospilus* (Hymenoptera: Tenthredinidae), a specialist feeder on *Salix* (Salicaceae) trees, and how it determines oviposition preference in lab and field conditions. We described the sequence of behaviors that lead to egg laying on the most and least preferred willow species. Then we studied the morphology of chemosensory structures present on the female antenna, cerci and ovipositor. Since phenolic glycosides (PGs) are the main secondary metabolites present in Salicaceae species, we investigated their role in host acceptance. We quantified these compounds in different willow species and correlated PG content with oviposition preference under lab and natural field conditions. We demonstrated a major role for contact cues in triggering *N. oligospilus* egg laying on the leaf surface of preferred willow genotypes. Firstly cues are sensed by antennae, determining to leave or stay on the leaf. After that, sensing is performed by abdominal cerci, which finally triggers egg laying. The lack of PGs in non-preferred species and the significant correlation observed between PGs, natural damage and oviposition preference suggest a role for these compounds in host selection. Our study suggests that in specialist feeders, secondary compounds normally acting as defenses can actually act as a susceptibility factor by triggering specific insect behavior for oviposition. These defensive compounds could be selected against to increase resistance.

## Introduction

Once an insect herbivore has located a plant by volatile and/or visual cues, the outer surface of leaves constitutes the zone of initial contact. The structural properties or chemical composition of the surface layer may then determine an insect’s acceptance of the plant as a host, and also provide a preference cue for specific localities such as the abaxial or adaxial leaf surface^1,2^. If herbivores can distinguish between host and non-host plants by epicuticular waxes or secondary compounds present on the leaf surface, they do not need to waste time and energy on unsuitable plants^3^. The chemical composition of the epicuticular leaf surface plays a role as oviposition stimulant for several insect orders. Waxy compounds such as long chain alkanes and alcohols have been shown to be oviposition stimulants in lepidopterans^4^ and dipterans^5^. Moreover, in several insect herbivores, epicuticular wax compounds act in synergism with other more polar secondary metabolites as oviposition stimulants. Studies have revealed that flavonoids, phenolic acids, glucosinolates, and sugars found on the plant surface can act as active principles in host recognition^6–10^.

Willow trees (*Salix* spp) belong to one of the principal genera of the Salicaceae family. In Argentina, willows are grown in the lower delta of the Paraná River, where 80% of the area corresponds to wetlands with unique ecological conditions, due to its water regime and biodiversity. Willows in this region have a high potential for use as paper pulp, wood based panels and sawn wood, which makes cultivation highly relevant for the economy of the area. This is why a breeding program is being carried out in order to find hybrid lines that meet the production requirements, such as a reduced susceptibility to pest insects (Teresa Cerrillo, INTA, personal communication). Phenolic glycosides (PGs) are a major group of secondary metabolites in the Salicaceae and have a key role in the interaction of willow trees with their herbivores. In the case of insect herbivores, most studies indicate that PGs function as feeding deterrents and reduce the fitness of generalist feeders^11–13^, while they can stimulate feeding^14^ and oviposition^15–17^ of specialist feeders.

The willow sawfly *Nematus oligospilus* Förster (Hymenoptera: Tenthredinidae), is a serious pest that feeds exclusively on species of *Salix* L. (Salicaceae) and has been reported worldwide^18–21^. This outbreaking species (however outbreaks are not very frequent) can cause damage up to 60% in steam diameter one year after defoliation^22^. Females of sawfly must find and recognize their host plant when they emerge as adults after pupating in the soil. Previous studies on *N. oligospilus* described differential oviposition preference depending upon willow genotype, and differences were registered in the chemical composition of the volatile emissions and the cuticular waxes between willow species with marked differences in oviposition preference^17,23^. Wax removal by gum Arabic treatment led to fewer eggs laid, suggesting a fundamental role of the surface chemistry during host recognition and acceptance^24^. Common alkane wax compounds have been reported in leaf extracts of preferred and non-preferred willow species. Since preferred leaf surfaces contained a more diverse wax profile including alcohols, acids and esters, a role of one or more of these common plant compounds during oviposition was suggested^24^. However, the influence of secondary metabolites characteristic to the Salicaceae family has not yet been investigated on leaves. Additionally, to our knowledge no study has characterized the chemical cues on willow leaves that could be perceived by *N. oligospilus* females and the associated behavioral response of the insect that leads to oviposition. Development of screening methods to identify host plant resistance to insects requires understanding the biology of the various phases of host-insect interaction as resistance can be manifested in a variety of different phenotypic traits that act at different points throughout the insect life cycle^25^. Identifying key plant traits that induce or prevent oviposition could provide opportunities to breed willow hybrids more resistant to *N. oligospilus*.

The behavioral sequence leading to egg laying or rejection of a potential host by a gravid female includes steps that allow contact chemoreceptors on appendages of its body to sample the chemical information available on the leaf surface, as was demonstrated for Lepidoptera^26^. This study aimed to answer firstly whether contact cues play a role during host recognition by *N. oligospilus* after landing on the leaf surface, and if so, we also aimed to find whether distinctive Salicaceae secondary metabolites (i.e. phenolic glycosides) were present on the leaf surface and could explain oviposition choice in *Salix* species of contrasting preference. We tested the hypothesis that the level of PGs can predict sawfly damage and oviposition. Thus we correlated PG level and oviposition preference in commercial willow hybrids. By studying the morphology of *N. oligospilus* appendages during oviposition behavior, we found evidence of the use of contact cues during the acceptance process once landed and before damaging the leaf surface to lay an egg. We also documented the presence of PGs in highly preferred species in leaves and on the leaf surface, and we found a correlation between PG content and oviposition preference in *Salix* species with different susceptibility. The implications of these results for *Salix* forestry are discussed.

## Methods and Materials

### Insects

Adult *N. oligospilus* females from a laboratory population were used in all bioassays. Every spring, the population was started from larvae and pupae collected in a field with a history of sawfly infestation in the lower Delta of the Paraná River (34° 10′ 23.08″ S, 58° 45′ 57.67″ W). Larvae were reared until pupation on fresh cut twigs of *Salix alba* L. × *S. alba* in transparent plastic boxes (46 × 30 × 32 cm) in a controlled environment chamber at 25 °C and L16:D8 hr photoperiod. Pupae were removed from the boxes and held in separate plastic jars until adult females emerged. During the growing season, the laboratory population was renewed several times with field-collected material to minimize selection for laboratory-adapted insects.

### Oviposition behavior, chemosensory structures and PG content in preferred and non-preferred willow species

#### Plants

Material from the National Program of Genetic Improvement for Salicaceae (willow genetic improvement area from Instituto Nacional de Tecnología Agropecuaria, INTA, Argentina) was used in this study. Although willows used for the experiments were grown during different years and under different conditions (i.e. in pots, field and semi-field conditions), previous studies have demonstrated that these different conditions do not change sawfly preference^17,23,27^. In addition, since PGs pattern is primarily genetically determined, these growing conditions did not affect the levels of PGs in leaves^28^. Natural colonization and feeding damage was evaluated during an outbreak of sawfly in 2008 in the Delta del Paraná area. Based on previous findings^17,24^ we selected two willow species of contrasting sawfly preference: the highly preferred *Salix nigra* (‘Alonzo nigra 4 INTA’) and the non-preferred *Salix viminalis* (‘mimbre macollado amarillo’), in which sawflies rarely lay eggs. For all laboratory experiments of this first part, during late winter of 2011, cuttings from *S. nigra* and *S. viminalis* were planted in consecutive rows, thus forming a nursery grown in the experimental station of INTA Castelar (34°36′29¨S, 58°40′10¨, Buenos Aires, Argentina). Plants were protected from insect damage (i.e. leaf-cutting ants) by placing ant baits in their surroundings.

#### Natural colonization and leaf damage

Feeding damage by *N. oligospilus* larvae was assessed in a field nursery by estimating defoliation of naturally colonized one year old plants. Plants corresponded to 16 trees descended from *S. nigra* subject to open pollination and 16 trees of a *S. viminalis* genotype. They were planted in rows of 10 or 20 and spaced by 1 m × 0.5 m, with a total of 32 plants. Level of damage was estimated on a scale of 6 levels: L0 = no defoliation, L1 ≤ 5% defoliation, L2 = 5–25%, L3 = 25–50%, L4 = 50–75%, L5 = 75–100%. Mann Whitney U-Test was performed in order to compare between plant species.

#### Oviposition behavior

We analyzed the oviposition behavior of sawflies elicited by both willow species in order to determine the key behaviors leading to egg laying, and the body parts involved in the chemical evaluation of the leaf surface. We recorded the sequence of behaviors, postures and actions that led finally to egg laying or leaf rejection. Females were released individually in the center of a Petri dish (14 cm diameter) bearing 2 leaves of either *S. nigra* (preferred) or *S. viminalis* (non-preferred). In the South Hemisphere females are parthenogenetic, thus, they are able to lay eggs as soon as they hatch. We observed the behavioral steps performed by each gravid female during 5 min after contacting the leaf surface. The variables quantified were: 1- Time spent on the leaf (total time during which the female contacted the leaf surface); 2- Number of females performing (number of females executing a given behavior) and 3- Percentage of time performing (time executing a given behavior divided by time spent on the leaf x 100). The variables were registered and quantified by means of JWatcher V1.0^29^ and statistically examined by Mann-Whitney *U-Test*.

In order to analyze the behavioral structure, stereotyped oviposition sequences were also identified and quantified. The procedure was similar to that described by Buteler *et al*.^30^ for the wheat stem sawfly. First, observations of all females were pooled by willow species. Then, the relative frequency of a behavior sequence was calculated as the total number of times that sequence was performed divided by the total amount of sequences recorded on each willow species. Comparisons were evaluated by Chi-square Test (χ^2^, P < 0.05).

#### Scanning Electron Microscopy (SEM) studies

Newly emerged females were preserved in 96% ethanol. The distal portion of their abdomen and entire antennae were dissected under a stereoscopic microscope (Olympus SZ61, Tokio, Japan), air-dried and mounted on stubs, coated with a gold-palladium (40–60%) or palladium alloy (Termo VG Scientific, West Sussex, England and Sputter Coater, Cressington Scientific Instruments, United Kingdom, respectively). Then, samples were observed by SEM at the Museo Argentino de Ciencias Naturales “Bernardino Rivadavia”, Argentina and at the Advanced Microscopies Center (Facultad de Ciencias Exactas y Naturales, Universidad de Buenos Aires, Argentina) (microscopes Philips XL 30, Eindhoven, The Netherlands, and Zeiss Supra 40, Germany, respectively). Microphotographs of possible chemosensory structures present in antennae, ovipositor and cerci were obtained.

#### Phenolic glycoside content

PGs were extracted by means of different treatments as detailed below:

Surface dipping: In order to screen for the presence of PGs, leaves were collected from the mid-section of 4 to 5 month-old plants and frozen at −20 °C in sealed plastic bags until use. Approx. 40 g of leaves from 3–5 individual plants were pooled and gently washed for 30 sec with 100 ml 100% methanol at room temperature. The dipping method was used only for comparative purposes. We did not intend to report total foliar content since the sawfly never penetrates the leaf beyond the first layer of epidermal cells^17^. Detailed procedure for PGs analysis is shown in Appendix S1 (Supporting information).

Gum arabic peeling: The presence of PGs within the epicuticular waxes of *S. nigra*, the most preferred species, was evaluated by analysis of surface waxes after mechanically removing them from the leaf. A 50% w/w aqueous solution of commercial gum Arabic (Biopack Productos Químicos, Argentina) was applied onto the entire leaf surface using a small paintbrush as in Badenes-Pérez *et al*.^31^. After 1 hr, the solution was dry and a thin polymer film could be gently peeled off in pieces, leaving the leaves physically intact (without damaging the epidermal tissue)^32^. Care was taken to ensure that no leaf tissue was removed with the gum Arabic peelings. Previous observations showed that the willow sawfly can lay eggs in both sides of the leaf^17^. Thus, ten leaves between 1 and 2 cm maximum leaf width were randomly chosen per plant (four plants total), five leaves for adaxial and five for abaxial peeling. The gum Arabic peelings for each of the leaves were weighed and later mixed with a double weight amount of HPLC grade water. Insoluble material was pelleted by centrifugation at 2500 g for 10 min, and the supernatants were used for PG analysis. Because of the very low concentrations of PGs in the gum Arabic peeling extracts, a very sensitive LC-MS/MS system was used for quantification. Analytes were chromatographed on an Agilent 1200 series HPLC system equipped with an Agilent XDB-C-18 column (4.6 × 50 mm, 1.8 um). Gradient elution with 0.05% aqueous formic acid (A) and acetonitrile (B) at a flow rate of 0.8 mL/min was applied using the following parameters: 5% B (0–0.5 min), 5–90% B (0.5–4.5 min), 90–100% B (4.5–4.52 min), 100% B (4.52–5.0 min), 5% B (5.1–8.5 min). Eluted compounds were ionized by electrospray (negative mode) and detected on an API 5000 mass spectrometer (Applied Biosystems, Carlsbad, CA, USA) using multiple reaction monitoring (MRM). MRM parameters for each analyte were optimized using standard compounds. Quantification was carried out by external standard curves for salicin (Alfa Aesar), salicortin, and tremulacin (both kindly provided by Dr. Bernd Schneider, MPI for Chemical Ecology, Jena, originally isolated by H. Thieme, Halle). MRM-parameters: (precursor ion m/z → product ion m/z; declustering potential [V], collision energy [V]): salicin (285 → 123; −50, −18), salicortin (423 → 123; −75, −30), tremulacin (527 → 123; −75, −34). In order to express the concentration of PGs per unit area, leaf area was measured. After removal of surface wax with gum Arabic, leaves were cut from the plant, pressed flat and the adaxial surface was photographed with a digital camera. The area of the leaf was determined by using ImageJ version 1.45 s software (National Institutes of Health, Bethesda, Maryland^33^). In order to determine the proportion of PGs present on the epicuticular surface, total PGs content in plant foliage was quantified. After the gum Arabic peeling, leaves were freeze-dried and ground to fine powder by agitation on a paint shaker (Skandex SO-10M, Fluid Management Europe, The Netherlands). A 10 mg portion of each sample was extracted with 1 ml methanol containing 0.8 mg/ml phenyl-β-glucopyranosid (Sigma Aldrich, St. Louis, MO, USA) as an internal standard. From this point, the extraction of phenolics followed the protocol for dipping extraction described in Appendix S1 (Supporting information).

### Natural colonization, oviposition preference and PGs content of commercial willow hybrids

#### Plants

Six genotypes, chosen due to their commercial value in the area, were compared: ‘Lezama’ (*S. matsudana × S. nigra*), ‘Yaguareté’ (*S. alba* × free pollination), ‘Los Arroyos’ (*S. matsudana* × *S. alba*), ‘Agronales’ (*S. matsudana* × *S. alba*), ‘Americano’ (*S. babylonica* var. sacramenta) and ‘Géminis’ (*S. matsudana* × free pollination).

#### Natural colonization and leaf damage

Feeding damage by *N. oligospilus* larvae was assessed in a field nursery in ¨Las Carabelas¨ field site (Buenos Aires, Argentina 34° 2′ 6¨S, 58° 6′ 7¨W) by estimating defoliation in naturally colonized 1 year-old plants. Plants were a mixture of 16 trees of each of the six genotypes above. They were planted in rows of 10 or 20 and spaced by 1 m × 0.5 m, with a total of 96 plants. Sawfly activity was monitored during an outbreak of sawfly from December 2007 to March 2008 in the Delta del Paraná area. The level of damage was estimated as explained in the previous section under the subtitle *Natural colonization and leaf damage*. Data were analyzed by Friedman test followed by LSM Multiple comparisons.

#### Oviposition preference bioassays

In late winter 2016, cuttings from different willow hybrids were planted individually in 5-l pots and kept outdoors under natural conditions. In order to establish a ranking for oviposition preference, dual-choice bioassays were carried out among all possible combinations during summer 2016. Bioassays were conducted inside the controlled environment chamber previously mentioned. Transparent plastic boxes (33 × 23 × 14 cm) containing a two-leaf fresh cut twig of each hybrid in tap water were employed. A newly emerged or 1day-old female was released in the center of the box and was allowed to lay eggs until death (normally up to 3 days). The number of eggs laid per genotype was recorded. In order to assign a quantitative value, oviposition preference was ranked for willow genotypes by normalized David’s score based on a paired comparison matrix of 6 × 6 (the six willow genotypes), which is the most appropriate dominance ranking method^34^. Calculations are shown in Appendix S2 (Supporting Information).

#### Analysis of PGs

In late winter 2016, 15 cuttings (30 cm length) of each willow hybrid were planted in a nursery in an experimental station in INTA Delta del Paraná (Buenos Aires, Argentina 34° 5′ 6¨S, 58° 22′ 23¨W). In order to compare PGs presence in surface dippings, leaves were collected from the mid-section of 4 to 5 month-old nursery plants and frozen at −20 °C in sealed plastic bags until use. Procedure was the same as described in the previous section (see *Phenolic glycoside content, surface dipping*). Afterwards, a correlation between PG content and the David’s score previously mentioned was performed by Pearson correlation (Infostat/E 2011).

#### Statistical analyses

Every test performed was conducted with the software package R 2.15.1 for Windows^35^ unless indicated.

## Results

### Oviposition behavior, chemosensory structures and PG content in preferred and non-preferred willow species

#### Natural colonization and leaf damage

**Figure 1:**
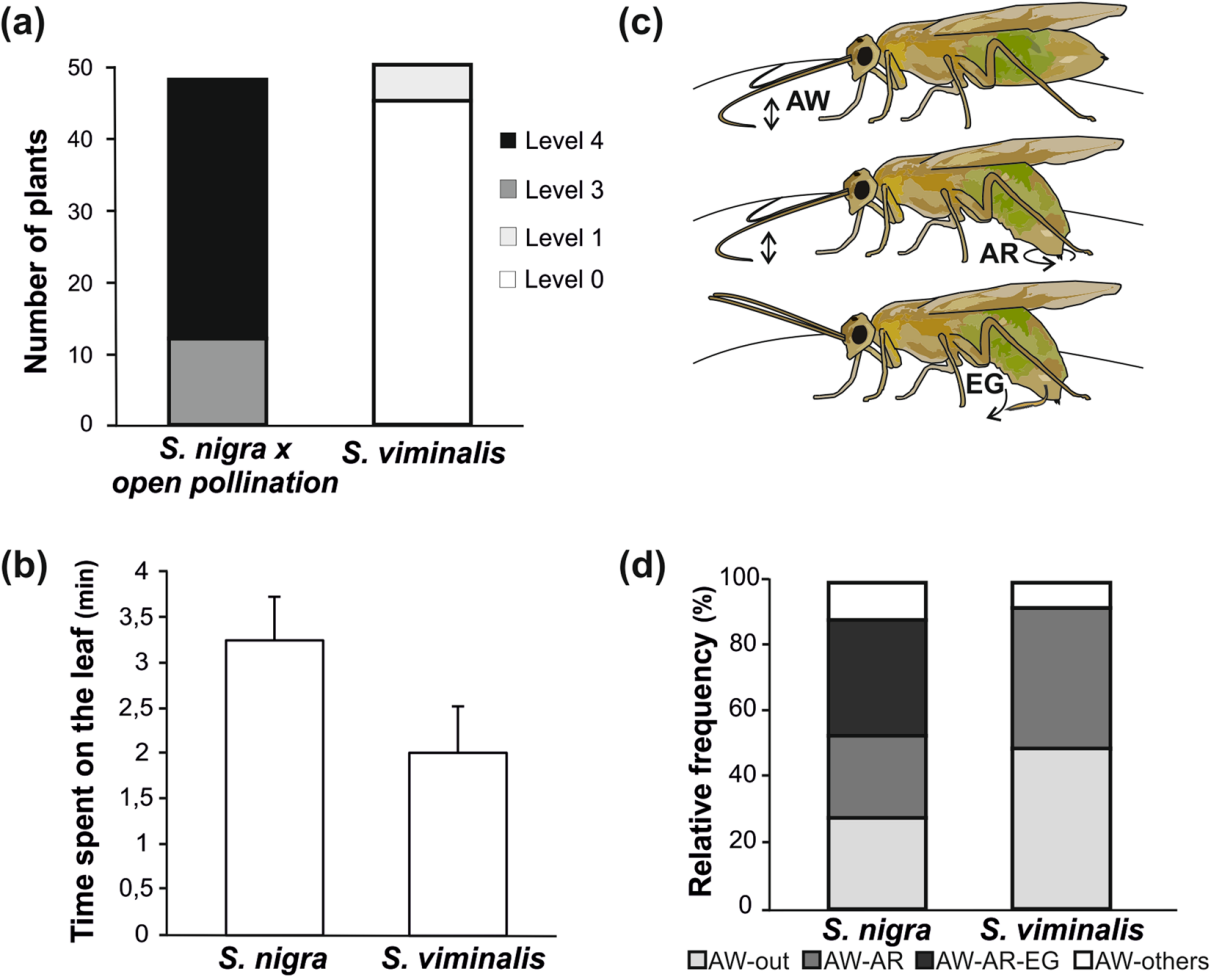
Natural colonization and oviposition behavior of *Nematus oligospilus* on *Salix* leaves. (**a)** Feeding damage in the field (Number of plants damaged). Mann Whitney U test P < 0.0001, N = 48 for *S nigra* and N = 50 for *S. viminalis* (**b**) Time spent by females on the leaf surface of *S. nigra* and *S. viminalis* during behavioral observations. Mann Whitney *U* test, NS, N = 12. (**c**) Scheme showing the three main behaviors performed by females on willow leaves (see description in Table 1). (**d**) Relative frequency of behavioral sequences (%) displayed by females once on the leaf surface of *S. nigra* (N = 68 sequences, 12 females) or *S. viminalis* (N = 59 sequences, 12 females). Chi square between *S. nigra* and *S. viminalis* was performed for each sequence showing significant differences for AW-out (p = 0.02), AW-BA (p = 0.03) and AW-EG-AR (p < 0.001) and non-significant differences for AW-others (p < 0.05).

Our field observations indicate that *S. nigra* genotypes were more damaged than *S. viminalis* genotypes (Mann Whitney U Test, *W* = 3576, P < 0.0001, N = 48 and N = 50 for *S. nigra* and *S. viminalis* respectively, Fig. 1a).

#### Oviposition behavior

Although there is a strong tendency suggesting that sawflies spent more time on *S. nigra* leaves, non-significant differences were found between *S. nigra* and *S. viminalis* (Fig. 1b, Mann-Whitney *U* test, P = 0.089, N = 12). A repertoire of six different behaviors associated with oviposition was identified: antennating while walking (AW), abdominal rubbing (AR), egg laying (EG), fanning (F), quiescent (Q) and antennal grooming (AG), described in Table 1. AW, AR and EG are schematized in Fig. 1c.

**Table 1:** Postures and behaviors performed by *Nematus oligospilus* gravid females after contacting a willow leaf surface.

Abbreviation	Behavior	Description
AW	Antennating while walking	Walking rapidly while tapping their antennae on the leaf surface
AR	Abdominal rubbing	The abdomen is bent into a comma shape, and the insect rubs the leaf surface with it while slowly walking and simultaneously antennating
EG	Egg laying	Females remain still with their ovipositor inserted into the leaf while laying the egg, antennae are immobile facing up
F	Fanning	Rapid wing movement without flying
Q	Quiescent	Quiescent females
AG	Antennal grooming	Grooming the antennae with forelegs

AW was identified as the first behavior that females do after contacting the leaf surface, and they resume this behavior immediately after doing any of the others. This is followed by AR, which can last more than one minute until selection of a suitable spot for ovipositor insertion. Once the ovipositor is inserted (EG), it may remain inside the tissue from a few seconds up to two minutes. Table 2 on the left shows the number of females that performed each of the previously mentioned behaviors on each willow species. The number of females carrying out AW on the leaves of both willow species was the same, while the behaviors AR and EG were more frequently observed on *S. nigra* (Chi Square Test p < 0.05). Egg deposition (EG) was never recorded on *S. viminalis* during the 5 min observation period. F, Q and AG were seldomly observed.

**Table 2:** Behavioral performance of *Nematus oligospilus*.

Behavior	Number of females performing		*χ*^2^	% Time performing		Mann Whitney
	*Salix nigra*	*Salix viminalis*		*Salix nigra*	*Salix viminalis*	
AW	12	11	ns	26.70 ± 5.89	59.82 ± 10.86	*
AR	10	5	*	43.31 ± 9.87	60.13 ± 9.77	ns
EG	10	0	*	37.31 ± 9.51	—	—
F	2	0	—	30.64 ± 1.53	—	—
Q	3	3	—	3.45 ± 1.28	72.16 ± 14.51	—
AG	2	1	—	0.85 ± 0.25	24.78	—

Number of females and percentage of time females performed a given behavior (mean ± SE) after contacting the leaf surface of either *Salix nigra* (N = 12) or *S. viminalis* (N = 11). Asterisk indicates significance with P < 0.05 and ‘ns’ indicates non-significant differences.

Table 2 on the right shows the time performing a given behavior divided by the total time on the leaf surface × 100 (% time). Females spent more time carrying out AW on *S. viminalis* and EG on *S. nigra*. Together, these observations suggest that sawflies evaluate the leaf surface by AW and, after finding an appropriate cue, they lower the abdomen searching for more cues.

Four stereotyped behavioral sequences were identified starting from AW, the action from which all other behaviors were initiated in every sequence. AW could be followed by leaving the leaf surface (AW-out), abdominal rubbing (AW-AR), abdominal rubbing followed by egg laying (AW-AR-EG) or another behavior such as fanning, quiescent or antennal grooming (AW-others). Figure 1d shows the relative frequency of the four previously mentioned sequences. In *S. viminalis*, AW-out and AW-AR were the most frequent sequences (49.15% and 42.37% respectively). On the contrary, in *S. nigra* the most frequent sequence was AW-AR-EG (35%), absent in *S. viminalis*. After AW, females seldomly performed another behavior such as fanning, grooming or becoming quiet (AW-other). Insertion of the ovipositor never occurred without previously rubbing the area with the abdomen, suggesting the existence of chemosensory structures associated with the ovipositor used to make decisions about oviposition choice. After laying an egg, females typically resumed AW. This sequence could be repeated over and over even on the same leaf.

#### Morphology of chemosensory structures

**Figure 2:**
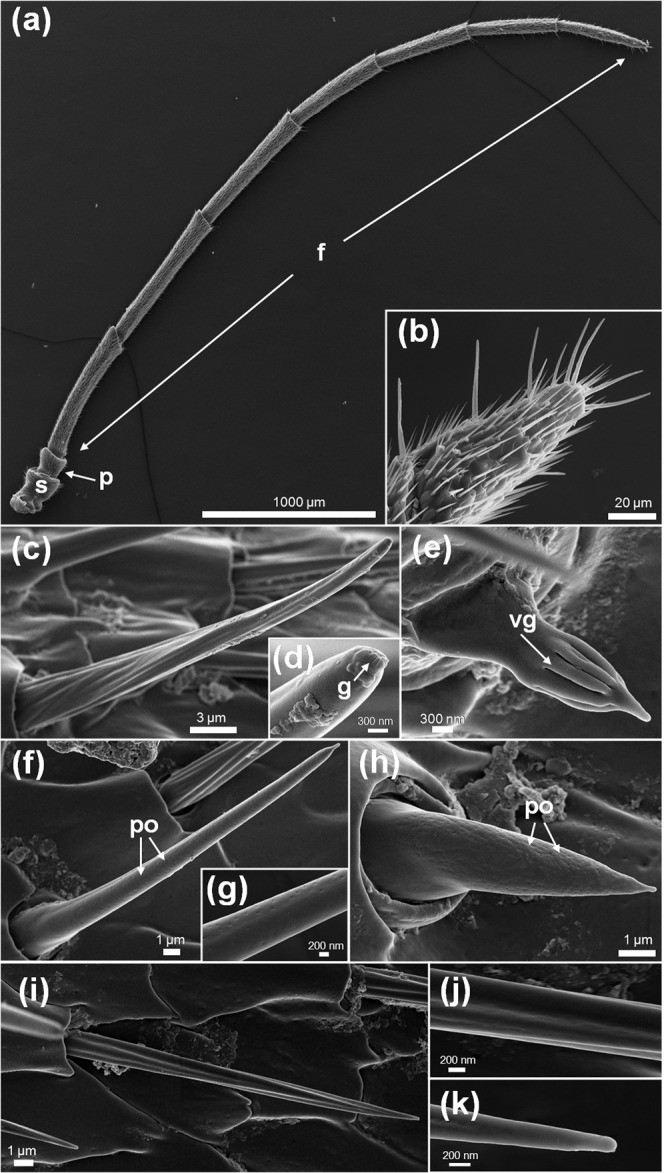
SEM of *Nematus oligospilus* antennae. (**a)** General view of filiform antennae, **(b)** Detail of distal flagellomere, **(c–h)** chemosensory sensilla, **(i–k)** mechanosensory sensillum. f = flagellum, h = groove, hv = vertical groove, s = scape, p = pedicel, po = pore.

*Nematus oligospilus* antennae are filiform and consist of (from proximal to distal end) a broad scape, a short triangular pedicel and a flagellum bearing 7 flagellomeres that gradually shortens towards the antennal tip (Fig. 2a,b). Based on their external morphology, SEM microphotographs revealed 4 types of chemosensory sensilla (Fig. 2c–h), and 1 possible mechanosensory sensillum (Fig. 2i–k). Among the first group, there are sensilla chaetica (20–60 μm length) bearing longitudinal striations on their surface and a single terminal pore, thus suggesting a contact chemoreception function (Fig. 2c,d). Besides, there are sensilla trichodea (22 μm length) (Fig. 2f,g) and sensilla basiconica (13 μm length) (Fig. 2h), both types with multiple pores on their cuticular walls suggesting a possible olfactory role. Finally, the fourth type was defined as a sensillum basiconica with grooves. Unlike all types previously mentioned, this one does not possess an articulated base and its length is considerably shorter (3 μm) (Fig. 2e).

**Figure 3:**
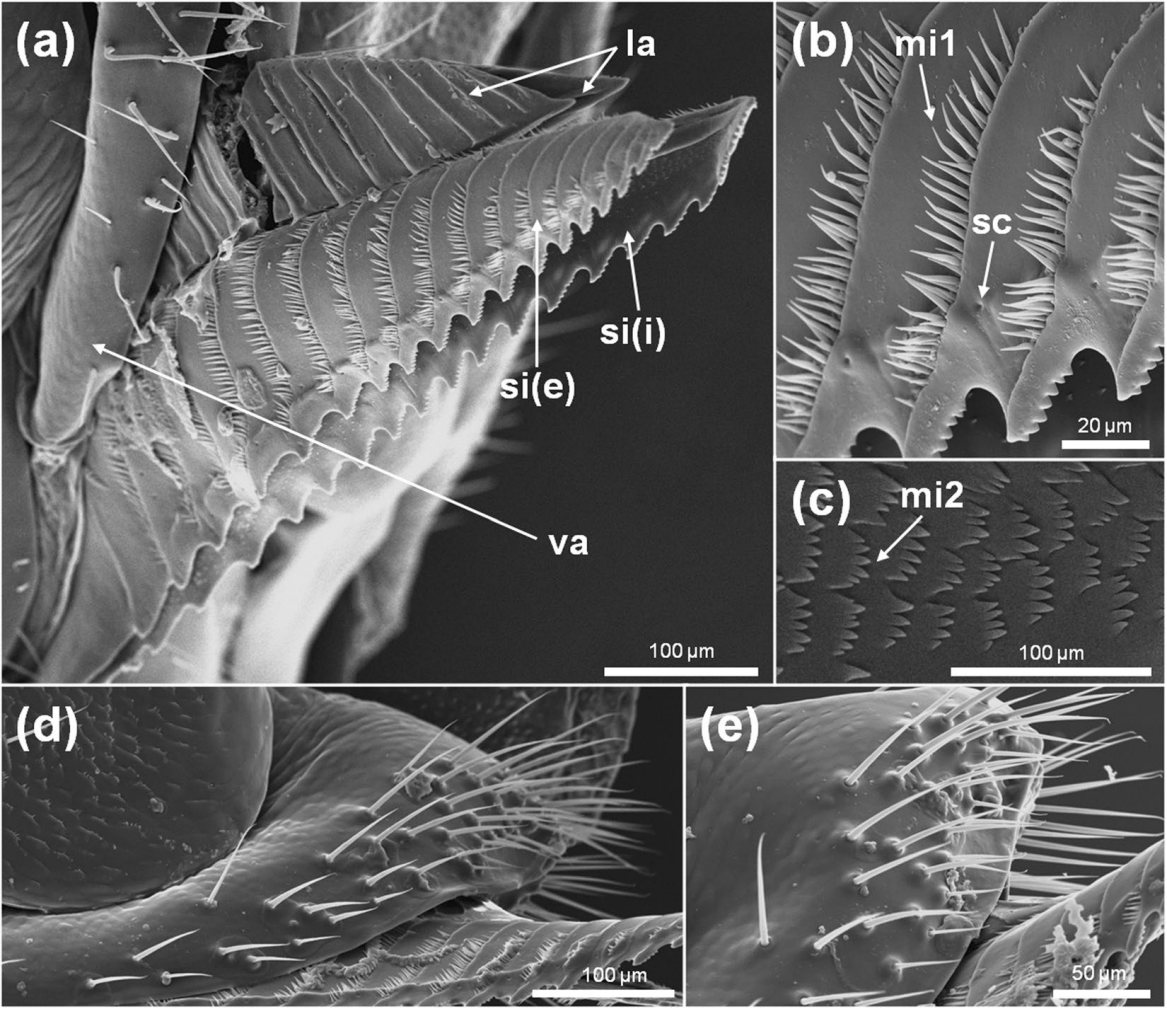
SEM of *Nematus oligospilus* ovipositor showing its structure and the presence of different types of mechanosensory sensilla. **(a)** Morphology of the saw and lance in ventrolateral view, **(b)** external face of the saw bearing campaniform sensilla and microtrichia type 1, **(c**) internal side of the saw revealing microtrichia type 2, **(d,e)** detail of the sheath in ventrolateral view, with bristle sensilla articulated at the base. sc = campaniform sensillum, la = lance, mi1 = microtrichia type 1, mi2 = microtrichia type 2, si(e) = external face of the saw, si(i) = internal face of the saw, va = sheath.

SEM microphotographs showed that the ovipositor consists of 3 pairs of valves (i.e. the saw, the lance and the sheath, from ventral to dorsal) (Fig. 3a), all of them bearing mechanosensory sensilla (Fig. 3b–e).

**Figure 4:**
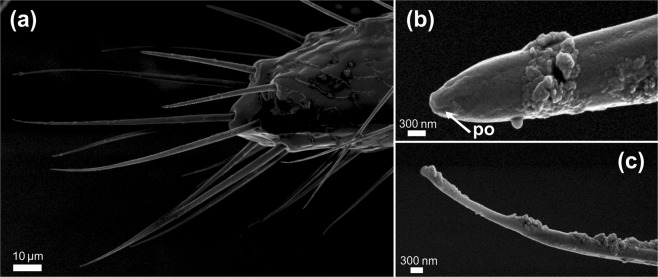
SEM of *Nematus oligospilus* cerci. (**a)** Morphology of the distal portion of the cercus, **(b)** Sensillum chaetica bearing a single terminal pore, **(c)** Bristle sensillum without any pores or grooves on its surface. po = pore.

A visual inspection of the cerci (Fig. 4a), articulated abdominal appendages of the 10th segment of the tergum, revealed the presence of two morphological types of sensilla. One type of sensillum chaetica (45 μm length) was present bearing a single terminal pore (Fig. 4b), thus probably related to contact chemoreception before oviposition. Also, bristle sensilla without pores or grooves (Fig. 4c) extended along the whole cercus surface, probably associated with mechanosensory stimuli.

### PG content

**Figure 5:**
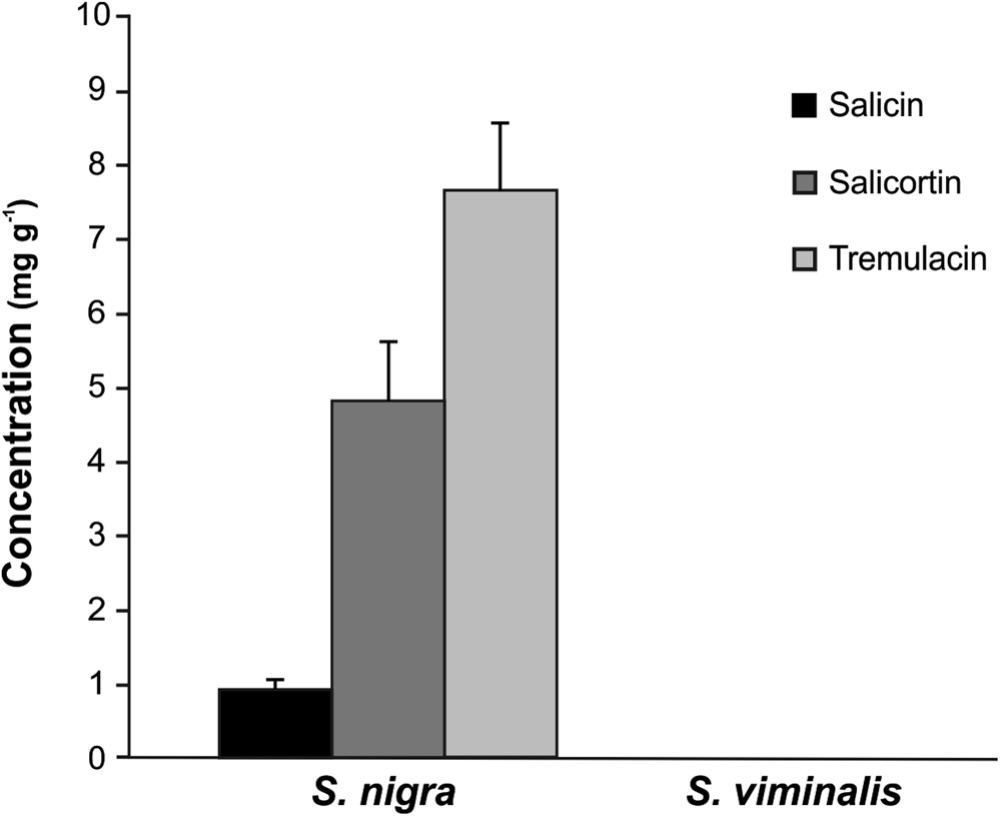
Quantification of phenolic glycosides (mass of analyte/mass of final extract DW, mean + SE) present in methanol surface dippings of *S. nigra* and *S. viminalis* leaves (N = 5).

The presence of the phenolic glycosides, salicin, salicortin and tremulacin, was quantified in methanol surface dipping extracts from *S. nigra* and *S viminalis* leaves. These metabolites were abundant in *S. nigra*, but were absent from *S. viminalis* extracts (limit of quantification between 0.30 and 0.70 mg/g for the different PGs, Fig. 5). Since willow sawfly females did not seem to damage the leaf surface before laying the egg (personal observation by light and SEM), we analyzed the possibility of PGs being on the epicuticular leaf surface as previously suggested for *Populus* spp.^36^. Data from Table 3 shows that PGs were present in similar amounts on gum arabic peelings of both the abaxial and adaxial epicuticular leaf surfaces of *S. nigra*. Their concentrations are low (0.03 to 0.29 ng/cm^2^) representing a maximum of 0.19% of the total PG content of the leaf.

**Table 3:** Phenolic glycosides found in gum arabic peelings from the abaxial and adaxial leaf surfaces of *Salix nigra* (n = 4).

	Abaxial		Adaxial	
	On surface (ng/cm^2^)	% of total foliar content	On surface (ng/cm^2^)	% of total foliar content
Salicin	0.04 ± 0.01	0.05 ± 0.01	0.03 ± 0.01	0.01 ± 0.00
Salicortin	0.22 ± 0.14	0.10 ± 0.06	0.24 ± 0.07	0.04 ± 0.02
Tremulacin	0.29 ± 0.21	0.19 ± 0.11	0.09 ± 0.02	0.02 ± 0.01

### Natural colonization, oviposition preference and PGs content of commercial willow hybrids

**Figure 6:**
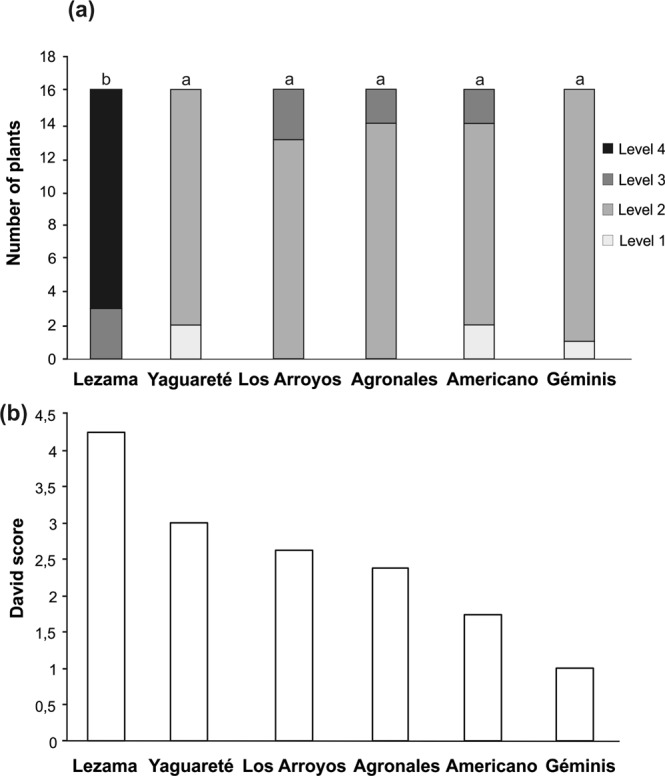
Natural colonization and oviposition preference in six different commercial willow hybrids. (**a)** Field survey: Level of damage by defoliation (Number of plants damaged). It was estimated by using the following scale: L0 = no defoliation, L1 ≤ 5% defoliation, L2 = 5–25%, L3 = 25–50%, L4 = 50–75%, L5 = 75–100%. Friedman test P < 0.0001, *indicates significant differences by LSM multiple comparisons, N = 16 for each genotype. (**b**) Laboratory assays: David’s score calculated from an oviposition preference matrix for *Nematus oligospilus* on different commercial hybrids (N = 90 comparisons, Landau’s Linearity Index h = 0.97; P = 0.02, see matrix in Table S1).

Our field observations on selected commercial willow hybrids showed differences in the temporal variation among genotypes from the beginning of the growing season (see Appendix S3, Supporting information). Sawfly damage starts at the beginning of the summer (i.e. December) only for ‘Lezama’, ‘Yaguarete’ and ‘Americano’ and reaches its highest levels in all genotypes by the end of the growing season (i.e. March). ‘Lezama’ (*S. matsudana* x *S. nigra*) genotypes were more damaged by larvae of *N. oligospilus* than the rest of the genotypes (Friedman test, P < 0.0001, LSM Multiple comparisons, P < 0.05 for ‘Lezama’. Fig. 6a). Oviposition preference in the lab was ranked for willow genotypes by normalized David’s score based on a paired comparison matrix of 6 × 6 (the six willow genotypes, Fig. 6b and Table S1). Landau’s Linearity Index was near 1 which indicates a hierarchy completely linear^37^. As expected from previous observations^17,24^ and these data, the willow hybrid containing *S. nigra* (‘Lezama’) was the most preferred, with the highest David Score, which means that it always received more eggs in the comparisons. ‘Géminis’ was the least preferred hybrid with a David Score of 1 (this hybrid was never preferred against the others).

**Figure 7:**
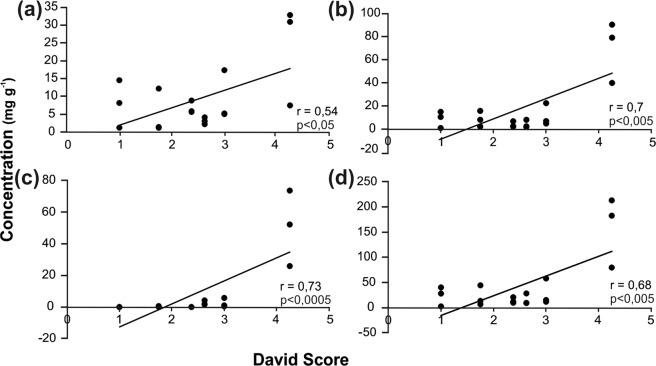
Correlation between David Score and concentration of PGs (**a**) Salicin. **(b)** Salicortin. (**c**) Tremulacin. (**d**) Total PGs. *r*: Pearson correlation coefficient and P value (N = 18). A trend line is also shown.

PGs content in dippings of the six commercial willow hybrids is shown in Table S2. Major PGs found were salicin, salicortin, HCH-salicortin and tremulacin. ‘Lezama‘ contained the highest concentration of all PGs. In the case of ‘Yaguareté’ and ‘Los Arroyos’, lasiandrin and an unknown PG (mass weight = 666) were also consistently detected but not quantified because of lack of standards. Interestingly ‘Lezama’ which possessed the highest David’s score, also contained the highest concentrations of all identified PGs. A further analysis showed that PG content is moderately strongly correlated with oviposition preference since a significant positive correlation between David Score and the presence of salicin, salicortin, tremulacin and total content of PGs was found (Fig. 7).

## Discussion

In our earlier work we showed that volatiles allow *N. oligospilus* females to identify appropriate host plant species^24^. Then a crucial second step carried out on leaves must be completed by the insects for oviposition. Once the female sawfly was on the leaf, the predominant behavioral sequences observed indicated that contact cues play a main role in triggering egg laying on preferred willow genotypes. Firstly cues are sensed by antennae, which seem to determine whether the insect leaves or stays on the leaf. When the insect makes the decision to stay, a second phase starts in which sensing is performed by abdominal cerci, which finally triggers egg laying. We characterized different types of chemosensilla from both the antennae and abdominal cerci of *N. oligospilus* females that support our observations on oviposition behavior. Natural oviposition preference under field and laboratory conditions was higher on foliage of *S. nigra*, which contain high PGs levels. The significant correlation observed between PGs and oviposition preference suggests a role of these compounds (or PGs derived metabolites) in host selection.

Our behavioral observations demonstrated that rubbing the leaf surface is triggered only after antennating while walking, and the decision to lay the egg is taken only after rubbing the leaf surface. Similar behaviors were reported by Buteler *et al*.^30^ in the wheat stem sawfly *Cephus cinctus*, where females did not oviposit without previous antennating and abdomen tapping. We propose that when assessing the leaf surface for oviposition, the acceptance process can be divided in stages^38^ and sawfly females only advance to the next stage when the appropriate cues are detected. At each stage, females employ sensilla from a different location: antenna, cerci, or ovipositor, which may help to gather sufficient information to make a more informed decision about oviposition. However, sawflies exposed to *S. viminalis* apparently did not find the appropriate cues to oviposit and so they continued assessing the host (AW, antennating while walking) without moving on to the next step. This response could be used to gain additional information, when dealing with limited information^39^. It is likely that in the field a female would abandon the non-preferred host after antennating without going to the next step, explaining why *S. viminalis* trees are seldomly damaged^24^, Fig. 1b).

The identification of different types of chemosensilla on the antennae and abdominal cerci of *N. oligospilus* females supports the observations on oviposition behavior. While females walk along the leaf surface, they exhibit antennae and ovipositor movements that suggest that these organs are in contact with cuticular waxes and secondary metabolites on the leaf surface. The evidence for chemosensilla on both antennae and abdominal cerci strongly suggest that females use them to evaluate the leaf surface chemical composition before deciding to lay eggs. Since there is no evidence that females injure the leaf surface before oviposition, we propose that gustatory receptors located in antennae and cerci enable detection of the surface chemicals on intact leaves. Choosing a suitable host plant is crucial for *N. oligospilus*, since neonate larvae have low mobility and develop mostly on the plant selected by their mother.

The preference performance hypothesis (or “mother knows best”) states that females will maximize their own fitness by laying eggs on plants where their offspring perform best (reviewed in^40^. Evidence for a preference-performance linkage has been found in many sawflies^41,42^; but see^43^). There is a strong relationship between oviposition preference and larval performance in *N. oligospilus* mediated by bottom-up effects of host plant vigor^27^ and induced defenses (personal observation). However a negative association between oviposition choices and larval performance was found in the interactions of other *Salix* species with sawflies, like *S nigra* and *S viminali*s that emit differential volatiles and have a different wax profile on leaf surfaces^17,24^.

The leaf surface is covered by various chemical compounds that can be used as cues for oviposition. The outer portion of the leaf cuticle is encrusted with waxes (i.e. intracuticular waxes) and a thin film of epicuticular waxes covers the outer surface^44^. The epicuticular crystals make up the predominant component of the wax and it is species-specific. Non-volatile secondary metabolites can also be deposited on the plant surface via diffusion from underlying cells and can be associated with waxes or stored in glandular trichomes^45,46^. The cuticular lipids together with more polar components are thought to constitute the major chemical signature influencing host plant recognition^44^, but these may have a synergistic effect with other secondary metabolites in stimulating oviposition^5,9,47–49^. We focused our chemical analysis of the willow leaf surface on low molecular weight phenolic glycosides (PGs), such as salicin and its derivatives because a link between oviposition preference and PGs has been proposed before for other specialist sawflies^14,16^. Particularly in the case of *N. oligospilus*, an association between salicin content and the number of eggs laid was already suggested by Braccini *et al*.^17^. Besides, *N. oligospilus* also prefers to oviposit on leaf surfaces with a high proportion of non-alkane wax chemicals^24^. However, the concentration of PGs does not fully explain oviposition preference since eggs can be found on *S. viminalis* even in the absence of PGs. Thus, we hypothesize that there might be a synergistic effect determining oviposition between wax compounds and PGs. This role of PGs and their potential synergistic effects with cuticular waxes needs to be tested in further bioassays.

*Nematus oligospilus* belongs to a primitive family within the Hymenoptera. The short adult life span of this sawfly (2–3 days) clearly dictates that oviposition sites should be found rapidly after eclosion. Indeed, females do not need mating or feeding before oviposition, and they hatch with most of their eggs ready to be laid. However, the host range of *N. oligospilus* is restricted to several *Salix* species. We found a simple stereotyped behavioral sequence leading to egg laying, and the presence of chemosensory receptors distributed along the antennae and cerci. These traits support the idea that in *N. oligospilus* a small number of chemoreceptors with great sensitivity to a few host-specific chemicals are the dominant factors in host selection^50^.

While this and many other studies have suggested the presence of secondary metabolites on the leaf surface act as kairomones, it has been difficult to establish procedures for unequivocally determining which compounds are on the plant surface and which are found only in underlying tissues. After rigorous determinations, we found low, but readily detectable quantities of PGs on the epicuticular leaf surface. These substances might be associated with stomata or the presence of trichomes as suggested by Reifenrath *et al*.^51^ and Badenes-Pérez *et al*.^31^. However, we cannot be certain that sawflies are able to sense such low quantities of PGs. Recently, imaging by MALDI mass spectrometry has been used to determine the fine-scale distribution of metabolites restricted to the leaf surface of *Arabidopsis thaliana* without interference from underlying tissues. These measurements revealed glucosinolates at biologically significant concentrations both, in the upper and lower leaf surfaces^10^. In the case of Salicaceae, salicortin and HCH-salicortin have been found on the leaf surface of *Populus deltoides* by leaf spray ionization technique^36^. These authors claimed that volatile products of enzymatic conversion of the PGs present on the leaf surface (salicylaldehyde and 6-Hydroxycyclohex-2-en-1-one, 6-HCH) may also serve as chemical signals for insects. Thus PGs, or some of their derived metabolites, could be part of the chemical signature that attracts this specialist insect.

Besides the chemical information provided, our results may have implications for agriculture or forestry. In order to reduce herbivore damage, it is desirable to develop plant genotypes that are intrinsically more resistant (i.e. direct defences) or less preferred to herbivores. The use of resistant plant varieties may be an important strategy to sustain wood and biomass production in willow tree plantations. Plant resistance in willows may act both directly on herbivores and indirectly through volatile emission to attract natural enemies of the herbivores (e.g.^52^). The present study showed that specific PGs can be used as cues for oviposition by the sawfly *N. oligospilus*, an important specialist insect pest on willow trees^22^. As a consequence, willows lacking PGs on their leaf surface may not be recognized as appropriate hosts. By correlating leaf PGs to the response of the willow sawfly, we determined the importance of PGs in contributing to susceptibility to the sawfly. Since it is widely known that plant chemistry is inherited from parental to hybrids^53^, one of the species examined was the hybrid ‘Lezama’ (*S. matsudana* x *S. nigra*). This taxon also had high PG levels and not coincidentally maintained the same susceptibility to the willow sawfly, as one of its parents (*S. nigra*). Conversely, those hybrids without PGs (i.e. *S. viminalis*) were not preferred by *N. oligospilus* for oviposition in natural field conditions and laboratory. Our study suggests that in the case of a specialist herbivore, secondary compounds normally acting as defenses are actually acting as a susceptibility factor. Thus, for the plant to evade such a specialist, one strategy is to lose the compounds. PGs are a trait that should be considered in willow breeding programs, in this case by being selected against. Exploiting knowledge on the chemical defenses of plants against pest insects provides opportunities for more sustainable resistance breeding.

## Supplementary information

Appendix S1 to S3 and tables S1 and S2
