# Author Correction: The use of Leaf Surface Contact Cues During Oviposition Explains Field Preferences in the Willow Sawfly *Nematus Oligospilus*

**DOI:** 10.1038/s41598-020-59356-x

**Published:** 2020-02-12

**Authors:** Patricia C. Fernández, Celina L. Braccini, Camila Dávila, Romina B. Barrozo, M. Victoria Coll Aráoz, Teresa Cerrillo, Jonathan Gershenzon, Michael Reichelt, Jorge A. Zavala

**Affiliations:** 1 Consejo Nacional de Investigaciones Científicas y Tecnológicas, Buenos Aires, Argentina; 2 INTA, Estación Experimental Agropecuaria Delta del Paraná. Paraná de las Palmas y Cl Comas s/n (2804), Campana, Buenos Aires, Argentina; 3 Universidad de Buenos Aires, Cátedras de Química de Biomoléculas y Bioquímica, Facultad de Agronomía. Av. San Martín 4453, C1417DSE Ciudad Autónoma de Buenos Aires, Argentina; 4 INTA, Instituto de Recursos Biológicos, Centro de Investigación de Recursos Naturales, De los Reseros y Dr. Nicolás Repetto s/n (1686), Hurlingham, Buenos Aires Argentina; 5 Universidad de Buenos Aires, Departamento Biodiversidad y Biología Experimental, Facultad de Ciencias Exactas y Naturales, Instituto Biodiversidad y Biología Experimental y Aplicada, CONICET - UBA, Ciudad Autónoma de Buenos Aires, Argentina; 6 PROIMI-CONICET Biotecnología, Av. Belgrano y Pje, Caseros (T4001 MBV), Tucumán, Argentina and Facultad de Ciencias Naturales e IML, UNT, Miguel Lillo 205, San Miguel de Tucumán, Argentina; 7 Max Planck Institute for Chemical Ecology, Jena, Germany

Correction to: *Scientific Reports* 10.1038/s41598-019-41318-7, published online 20 March 2019

The original version of this Article contained an error in the title, where “*Nematus oligospilus”* was incorrectly given as “*Nematus Oligospilus*”.

In addition, in Fig. 2, panel (b) was a duplication of part of panel (a).

These errors have now been corrected in the PDF and HTML versions of the Article.

