# Supplementary material for: The use of Leaf Surface Contact Cues During Oviposition Explains Field Preferences in the Willow Sawfly Nematus oligospilus
Source: Sci Rep. 2019 Mar 20;9:4946. doi: 10.1038/s41598-019-41318-7 (PMC6426852; doi:10.1038/s41598-019-41318-7)
Supplement: Supplementary file 1 — Appendix S1 to S3 and tables S1 and S2 [file 41598_2019_41318_MOESM1_ESM.pdf]

“The use of leaf surface contact cues during oviposition explains field preferences in the willow sawfly *Nematus oligospilus*”. Scientific Reports. Patricia C. Fernández, Celina L. Braccini, Camila Dávila, Romina B. Barrozo, Maria Victoria Coll Aráoz, Teresa Cerrillo, Jonathan Gershenzon, Michael Reichelt, Jorge A. Zavala. INTA-CONICET-UBA, pcfernan@agro.uba.ar

Appendix S1: PGs analysis after dipping extraction (SuppInfo)

Methanol from the extracts was evaporated to dryness under nitrogen. Dried samples were re-dissolved in 1 ml 100% methanol containing 0.8 mg/ml phenyl-β-glucopyranosid (Sigma Aldrich, St. Louis, MO, USA) as an internal standard, and incubated on horizontal shaker for 10 min at room temperature. After centrifugation at 14000 rpm for 10 min, 300 µl of the supernatant was mixed with 300 µl distilled H_2O before analysis. Samples were analyzed with a high-performance liquid chromatograph, HPLC (Agilent, Series 1100, Santa Clara, CA, USA), equipped with a diode array detector, DAD (G1315B). A reverse-phase (RP) C18 column (EC 250/4.6 Nucleodur Sphinx, RP5 µm, Macherey-Nagel, Düren, Germany) was used for the separation of the compounds using conditions described in Boeckler et al. (2016). Concentrations were calculated on the basis of the peak areas at 200 nm relative to the internal standard applying experimentally determined response factors. Weight based response factors relative to the internal standard phenyl-β-glucopyranosid were determined using the following standards: salicin (Alfa Aesar), salicortin, HCH-salicortin and tremulacin (all three kindly provided by Dr. Bernd Schneider, MPI for Chemical Ecology, Jena, originally isolated by H. Thieme, Halle, (Thieme and Benecke 1971). Response factors are the following: salicin, 0.45; salicortin, 0.87; HCH-salicortin, 0.65; and tremulacin, 0.44. Results of content of PGs were relativized to dry weight of the extract.

References

Thieme H and Benecke R (1971) Die Phenolglykoside der Salicaeen, 8, Mitteilung: Untersuchung über die Glykosidakkumulation in einigen mitteleuropäischen Populus-Arten; Pharmazie 26:227–231

“The use of leaf surface contact cues during oviposition explains field preferences in the willow sawfly *Nematus oligospilus*”. Scientific Reports. Patricia C. Fernández, Celina L. Braccini, Camila Dávila, Romina B. Barrozo, Maria Victoria Coll Aráoz, Teresa Cerrillo, Jonathan Gershenzon, Michael Reichelt, Jorge A. Zavala. INTA-CONICET-UBA, pcfernan@agro.uba.ar

Appendix S2: Willow genotype ranking calculations (SuppInfo)

In order to assign a qualitative value which represents the magnitude preference over the other willows, the oviposition preference of sawflies over different willow genotypes was ranked based on a paired comparison matrix according to Pirk and López de Cassenave (2011). The paired comparison matrix was 6 x 6 (the six willow genotypes), with each cell *ij* representing a pair of willow genotypes, and its value, the number of times the willow genotype *i* (row) was preferred to the willow genotype *j* (column) in all trials (3 repetitions) involving *i* and *j*. Differences in number of eggs resulting from the *N. oligospilus* oviposition preference between pairs of willows were tested by using a Chi-square test (χ^2, P<0.05). In case of significant differences, the value assigned to an *ij* cell was 1 for the preferred and 0 for the non-preferred willow genotype. For combinations in which the oviposition preference in a trial was not contrasting (χ^2, P >0.05), a value of 0.5 was assigned to both genotypes, because this situation was considered a tie. Hierarchy was verified by Landau's linearity index, *h* (Landau 1951). When all the elements in a paired comparisons design can be arranged into a hierarchical order, the hierarchy is linear (Pirk and Lopez de Cassenave 2011). The Laundau's index ranges from 0 to 1, where 1 indicates complete linearity and 0 indicates that all species are equally preferred. The significance of the linearity index *h* was determined by means of a randomization test (de Vries 1995). Finally, the oviposition preference was ranked for willow genotypes by normalized David's score, which is the most appropriate dominance ranking method (Gammell et al. 2003). This score equally reflects the strength of non- preferred species by species *i* and the weakness of species by which species *i* was defeated (de Vries 1998). It varies between 0 and *N-1*, where *N* is the number of compared species, with higher values indicating higher preference levels.

References

Gammell MP, Vries HD, Jennings DJ, Carlin CM, Hayden TJ (2003) David's score: a more appropriate dominance ranking method than Clutton-Brock et al.'s index. Animal behaviour 66:601-605.

Landau H (1951) On dominance relations and the structure of animal societies: I. Effect of inherent characteristics. The bulletin of mathematical biophysics 13:1-19.

Pirk GI, López de Casenave J (2011) Seed preference of three harvester ants of the genus *Pogonomyrmex* (Hymenoptera: Formicidae) in the Monte desert: are they reflected in the diet? Ann Entomol Soc Am 104:212-220.

Vries HD (1995) An improved test of linearity in dominance hierarchies containing unknown or tied relationships. Animal Behaviour 50:1375-1389.

Vries HD (1998) Finding a dominance order most consistent with a linear hierarchy: a new procedure and review. Animal Behaviour 55:827-843.

“The use of leaf surface contact cues during oviposition explains field preferences in the willow sawfly *Nematus oligospilus*”. Scientific Reports. Patricia C. Fernández, Celina L. Braccini, Camila Dávila, Romina B. Barrozo, Maria Victoria Coll Aráoz, Teresa Cerrillo, Jonathan Gershenzon, Michael Reichelt, Jorge A. Zavala. INTA-CONICET-UBA, pcfernan@agro.uba.ar

Appendix S3: Temporal variability of the natural colonization in six different commercial willow hybrids (Suppinfo). Level of damage by defoliation (Number of plants damaged) was estimated from December 2007 to March 2008 by using the following scale: L0 = no defoliation, L1 ≤ 5% defoliation, L2 = 5 - 25%, L3 = 25 - 50%, L4 = 50 - 75%, L5 = 75 - 100%, N=16 for each genotype. **(a)** ‘Lezama’ (*S. matsudana* × *S. nigra*), **(b)** ‘Yaguareté’ (*S. alba* × open pollination), **(c)** ‘Los Arroyos’ (*S. matsudana* × *S. alba*), **(d)** ‘Agronales’ (*S. matsudana* × *S. alba*), **(e)** ‘Americano’ (*S. babylonica* var. sacramenta) and **(f)** ‘Géminis’ (*S. matsudana* × open pollination).

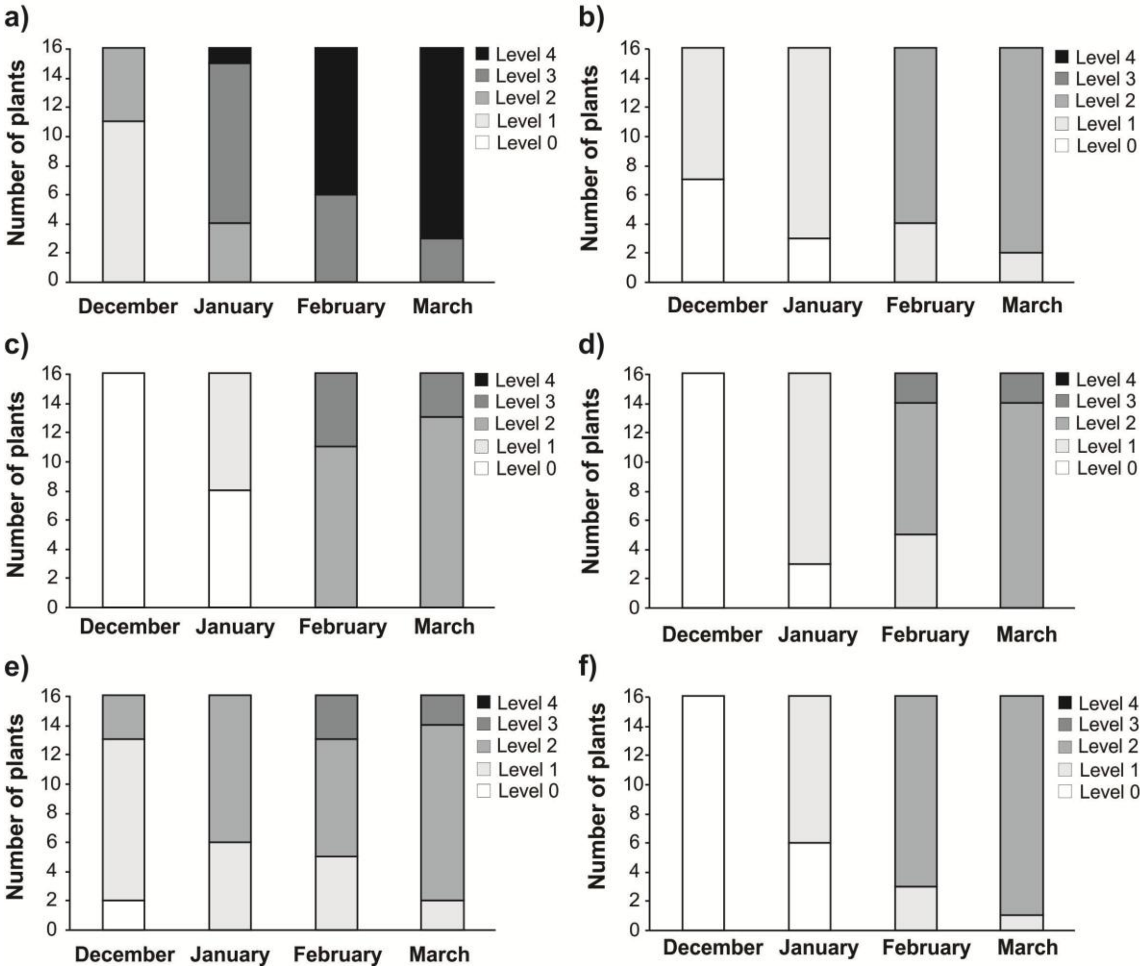

“The use of leaf surface contact cues during oviposition explains field preferences in the willow sawfly *Nematus oligospilus*”. Scientific Reports. Patricia C. Fernández, Celina L. Braccini, Camila Dávila, Romina B. Barrozo, Maria Victoria Coll Aráoz, Teresa Cerrillo, Jonathan Gershenzon, Michael Reichelt, Jorge A. Zavala. INTA-CONICET-UBA, pcfernan@agro.uba.ar

Table S1. Preference matrix for *Nematus oligospilus* on different commercial genotypes (N=90 comparisons) and David Score.

	Preference matrix						David's
	Lezama	Yaguareté	Los Arroyos	Agronales	Americano	Géminis	score
Lezama		3	3	2.5	3	3	4.3
Yaguareté	0		2	2	2.5	3	3
Los Arroyos	0	1		2	3	2	2.6
Agronales	0.5	1	1		1.5	3	2.4
Americano	0	0.5	0	1.5		2.5	1.8
Géminis	0	0	1	0	0.5		1

Landau´s Linearity Index h = 0.97; P = 0.02

Table S2. Phenolic glycosides (PGs) content (mg/g extract DW, mean ± SE) in six different commercial *Salix* hybrids (N=3).

Salix genotype	*matsudana x nigra*	*alba x open pollination*	*matsudana x alba*	*matsudana x alba*	*babylonica*	*matsudana x open pollination*
Common name	´Lezama´	´Yaguareté´	´Los Arroyos´	´Agronales´	´Americano´	´Géminis´
Salicin	23.7 ± 6.7	9.2 ± 3.3	3.2 ± 0.5	6.8 ± 0.8	5.0 ± 3.0	8.0 ± 3.1
Salicortin	69.7 ± 12.4	11.5 ± 4.5	4.2 ± 1.6	4.2 ± 1.2	8.8 ± 3.2	8.9 ± 3.4
HCH-salicortin	14.4 ± 3.3	5.2 ± 2.9	5.7 ± 2.5	3.4 ± 0.8	7.5 ± 3.3	6.7 ± 2.7
Tremulacin	50.6 ± 11.3	2.4 ± 1.4	2.5 ± 0.7	0.0 ± 0.0	0.2 ± 0.2	0.0 ± 0.0
Lasiandrin	-	++	+	-	-	-
Unknown MW=666	-	++	+	-	-	-
